# Reliability and Validity of the New Tanaka B Intelligence Scale Scores: A Group Intelligence Test

**DOI:** 10.1371/journal.pone.0100262

**Published:** 2014-06-18

**Authors:** Yota Uno, Hitomi Mizukami, Masahiko Ando, Ryoji Yukihiro, Yoko Iwasaki, Norio Ozaki

**Affiliations:** 1 Department of Child and Adolescent Psychiatry, Nagoya University Graduate School of Medicine, Nagoya, Japan; 2 Department of Psychology, Chiba Juvenile Detention Home, Chiba, Japan; 3 Center for Advanced Medicine and Clinical Research, Nagoya University Hospital, Nagoya, Japan; 4 Department of Psychology, Kyoto Gakuen University, Kyoto, Japan; 5 Department of Psychology, Saitama Juvenile Detention Home, Saitama, Japan; Tokyo Metropolitan Institute of Medical Science, Japan

## Abstract

**Objective:**

The present study evaluated the reliability and concurrent validity of the new Tanaka B Intelligence Scale, which is an intelligence test that can be administered on groups within a short period of time.

**Methods:**

The new Tanaka B Intelligence Scale and Wechsler Intelligence Scale for Children-Third Edition were administered to 81 subjects (mean age ± SD 15.2±0.7 years) residing in a juvenile detention home; reliability was assessed using Cronbach’s alpha coefficient, and concurrent validity was assessed using the one-way analysis of variance intraclass correlation coefficient. Moreover, receiver operating characteristic analysis for screening for individuals who have a deficit in intellectual function (an FIQ<70) was performed. In addition, stratum-specific likelihood ratios for detection of intellectual disability were calculated.

**Results:**

The Cronbach’s alpha for the new Tanaka B Intelligence Scale IQ (BIQ) was 0.86, and the intraclass correlation coefficient with FIQ was 0.83. Receiver operating characteristic analysis demonstrated an area under the curve of 0.89 (95% CI: 0.85–0.96). In addition, the stratum-specific likelihood ratio for the BIQ≤65 stratum was 13.8 (95% CI: 3.9–48.9), and the stratum-specific likelihood ratio for the BIQ≥76 stratum was 0.1 (95% CI: 0.03–0.4). Thus, intellectual disability could be ruled out or determined.

**Conclusion:**

The present results demonstrated that the new Tanaka B Intelligence Scale score had high reliability and concurrent validity with the Wechsler Intelligence Scale for Children-Third Edition score. Moreover, the post-test probability for the BIQ could be calculated when screening for individuals who have a deficit in intellectual function. The new Tanaka B Intelligence Test is convenient and can be administered within a variety of settings. This enables evaluation of intellectual development even in settings where performing intelligence tests have previously been difficult.

## Introduction

If delayed intellectual development in a child remains unnoticed and proper care is not received in a timely manner, maladjustment in society, loss of self-esteem, and behavioral problems may occur [1]–[7]. In fact, many published reports have suggested a high prevalence of deficit in intellectual function in offenders [8]–[10]. Therefore, in child-rearing and educational settings, providing services adjusted to the cognitive characteristics of a child, including intellectual development, is important. In addition, from a point of social safety, it is also desirable to provide specific approaches to offenders with intellectual disability (ID) that reflect their intellectual development in order to reduce recidivism [11]–[13]. Therefore, individually assessing intellectual development adequately and with flexibility in many settings is desired.

The Wechsler Intelligence Scale for Children (WISC) [14] is commonly used for intelligence testing. The WISC uses special test equipment, is administered on individuals, and in addition to overall intelligence, it can assess abilities in several domains, including verbal and performance IQ. Testing requires approximately 1–2 hours, with a trained examiner administering all testing materials. A shorter version of the WISC [15], which uses certain subtest items to estimate overall intellectual development, is available. However, the short form is similar to the full test in that it can only be performed on individuals, and requires special test equipment as well as experience in administering the test. Therefore, in settings where there are many individuals suspected of having ID, but a relative lack of specialists in ID or mental health, such as in justice facilities, it is impractical to perform individual intelligence tests on individuals within an entire group. Consequently, convenient intelligence tests or simple screening scales become more attractive.

On the other hand, intelligence tests administered on groups of individuals are available. To our knowledge, there are several group tests which have been standardized in English-speaking countries [16]–[21], but only a few tests exist outside English-speaking countries. One of which is the new Tanaka B Intelligence Scale [22]. Testing may be conducted simultaneously on groups, with no special equipment required, and only writing materials, test paper, and a short time (only 40–45 minutes) frame are required. This is convenient for assessing overall intellectual development. This test seems to be suitable for individuals with varied educational backgrounds, in varied linguistic environments, and over a wide range of linguistic levels, because it does not need complex instructions and is easily understood. This test was originally developed by Kanichi Tanaka in 1936 and has repeatedly been revised and restandardized since the 1930s. The test was most recently restandardized in 2001–2002 and has very high split-half correlations (*r* = 0.89 to 0.96) and high test-retest reliability (*r* = 0.73 to 0.79). There is high validity (*r* = 0.69 to 0.78) with overall scholastic ability, including Japanese language, mathematics, science, social studies, and English [22].

However, academic performance is influenced not only by intellectual development, but also by various environmental factors, including educational background. To the best of our knowledge, the correlation between the new Tanaka B Intelligence Scale and other intelligence tests has not yet been investigated. Therefore, more information about the reliability and validity of this test is needed along with a standardized test to assess individual intellectual development.

Thus, the present study examined the reliability of the new Tanaka B Intelligence Scale and its concurrent validity with the Wechsler Intelligence Scale for Children-Third Edition (WISC-III), which is one of the most-used tests to assess the intelligence quotient of individuals. Moreover, the clinical utility of the new Tanaka B Intelligence Scale as a screening scale for individuals who have a deficit in intellectual function [WISC-III full intelligence quotient (FIQ) less than 70] was evaluated. If the new Tanaka B Intelligence Scale is standardized, intellectual assessment becomes possible even in settings where the number of cases who can receive individual assessment has previously been limited, such as schools and correctional facilities. This will contribute to setting goals for those individuals and planning strategies to achieve those goals.

## Materials and Methods

### New Tanaka B Intelligence Scale

The new Tanaka B Intelligence Scale [22], [23] uses diagrams, pictures, and symbols (such as numbers), and has no tasks or problems presented in story form. Diagrams and symbols are used for responses, so testing is not readily affected by learning differences, such as reading or writing, or by linguistic or cultural influences. There are seven subtest items, including mazes, calculating cubes, replacing figures and numbers, difference discrimination of character strings, completing a number series, erasing figures, and completing figures. Testing is divided into five parts depending on the subject’s age, including testing for ages 6–8 years old (early elementary school), ages 8–10 years old (middle elementary school), ages 10–12 years old (late elementary school), ages 12–14 years (junior high school years 1 and 2), and age 14-adult (junior high school year 3 and high school and up). Testing was performed for subjects aged ≥14 years in the present study.

### Procedures and Subjects

Of the 81 children/adolescents in a juvenile detention home between January 1, 2009 and December 31, 2010, all took both the new Tanaka B Intelligence Scale and the WISC-III. One juvenile detention home is generally located in each prefecture. These are public facilities where children/adolescents from age 12 to less than 20 years who have committed a criminal act in a prefecture are detained for a maximum of eight weeks until a court hearing and for the purpose of evaluating the individual and deciding a future educational plan. The homes are administered by the Correction Bureau of the Ministry of Justice in Japan.

Individuals who are detained at any juvenile detention home in Japan take a test battery which is prescribed by the Correction Bureau to assess their abilities and needs within three days when they enter a home. If it is determined that additional tests need to be performed, each home can perform them at its discretion. The new Tanaka B Intelligence Scale, some personality tests and so on are contained in the battery. It is conducted in a party of three to fifteen people. The home in question performs the Wechsler Intelligence Scale in addition to the battery at its own discretion, because most tests in the battery have not been standardized, yet. The WISC-III was performed individually by a psychologist who was not same tester who examined the new Tanaka B Intelligence Scale between the day after the group test and a judgment. Motivations of the cases for all the tests (group tests and individual tests) were high, because their attitude during the tests is reflected in their court.

Although some subjects had multiple admissions to the juvenile detention home, testing was not performed on the second admission or thereafter. In other words, no subject was enrolled in the study more than once.

The mean age of the subjects was 15.2 (SD 0.7) years, with a range of 14.0 years to 16.8 years; 77 (95.1%) subjects were male, and four (4.9%) were female. Regarding intellectual development, the mean WISC-III FIQ was 76.5 (SD 15.0), with a range from 51 to 127. There were 58 individuals who had an FIQ<85 (71.6%), of which 26 individuals had an FIQ<70 (32.1%). However, no subject had been detected having a deficit in intellectual function prior to testing. There were absolutely no subjects who received special services to aid intellectual development. Moreover, as well as 26 out of 81 cases having ID (FIQ less than 70), 5 cases had *Attention Deficit/Hyperactivity Disorder*, and 1 each respectively exhibited *Pervasive Developmental Disorder - Not Otherwise Specified*, *Conduct Disorder*, and *Somatoform Disorder*. There were no individuals who had more than two diagnoses. These diagnoses were determined based on the Diagnostic and Statistical Manual of Mental Disorders, Fourth Edition, by experienced child psychiatrists.

### Statistical Analysis

#### Power calculation

Power analysis was performed to establish the power needed to interpret the results for the present study. A SD of 15 was estimated for both the new Tanaka B Intelligence Scale IQ (BIQ) and the FIQ. Power was calculated using a 95% confidence interval (±5) of the difference between the two intelligence tests.

#### Internal consistency

To assess the internal consistency of the new Tanaka B Intelligence Scale, Cronbach’s alpha coefficient was calculated for the seven subtest items, including mazes, calculating cubes, replacing figures and numbers, difference discrimination, completing a number series, erasing figures, and completing figures.

#### Accuracy of the BIQ score for the FIQ score

The BIQ and FIQ scores were plotted, and the differences between them were determined. To assess deviation and accuracy of the BIQ for the FIQ, mean percentage error (MPE) and root mean squared error (RMSE) were calculated [24].

#### Validity and clinical utility

To assess concurrent validity of the new Tanaka B Intelligence Scale with the WISC-III, the IQ scores on both tests were examined using a one-way analysis of variance intraclass correlation coefficient (ANOVA ICC).

Next, the performance of the new Tanaka B Intelligence Scale as a screening scale was evaluated using receiver operating characteristic (ROC) curve analysis. Areas under the ROC curve (AUC) and their 95% confidence intervals (95% CIs) were calculated using the parametric method. In addition, the likelihood and post-test probability for detection of FIQ<70 for each BIQ stratum were calculated using the stratum-specific likelihood ratio (SSLR) [25].

The SSLR indicates the odds ratio and is calculated as the “proportion of persons with a positive test among those with a disorder” divided by the “proportion of persons with a negative test among those without the disorder.” The SSLR for each stratum is calculated as follows: SSLR = (n_1 g_/N_1_)/(n_0 g_/N_0_), where n_1 g_ is the weighted number of subjects with the disorder in the g^th^ stratum, N_1_ is the weighted total number of subjects with the disorder, n_0 g_ is the weighted number of subjects without the disorder in the g^th^ stratum, and N_0_ is the weighted total number of subjects without the disorder. The post-test probability is a function of the SSLR, pretest odds, and post-test odds and is calculated as follows: pretest odds × SSLR = post-test odds, and post-test probability = (post-test odds)/(1+ post-test odds) [26]. Therefore, if the SSLR = 1, then the discrimination accuracy of the test is equal to chance probability. The closer the SSLR is to greater than one, the higher the likelihood of having a disorder. The closer the SSLR is to less than one, the lower the likelihood of having a disorder.

The validity of a test has traditionally been assessed using a single cut-off point approach in terms of sensitivity and specificity. A drawback in this case is that values not meeting the cut-off point, even values of results obtained as continuous variables, are treated uniformly regardless of magnitude. When using the SSLR for values of results obtained as continuous variables, the values of the results are stratified, and the probability of a disorder within each stratum can be calculated.

### Ethical Considerations

The protocol of this study was approved by the Ethics Committees of the Japanese Association of Correctional Medicine and the Nagoya University Graduate School of Medicine, and the study itself was conducted in conformity with the established ethical standards of all institutions. All cases involved in this study had come out of the home. Furthermore, all of the data which was used in this study was clinical data obtained conventionally during the course of considering diagnosis and treatment, and we used it secondarily and retrospectively. Therefore, the requirement for informed consent was waived. Cooperation in the study placed no burden on individual cases. Personal information regarding subjects in this study and the resulting data were rendered anonymous, and analyses were performed using only quantitative data that could not be linked to any particular subject.

## Results

### Power Calculation and Internal Consistency

The power for the present study including the 81 subjects was 0.85. Cronbach’s alpha coefficient for the seven BIQ subtest items was α = 0.86, indicating high internal consistency.

### Accuracy of the BIQ Score for the FIQ Score

**Figure 1:**
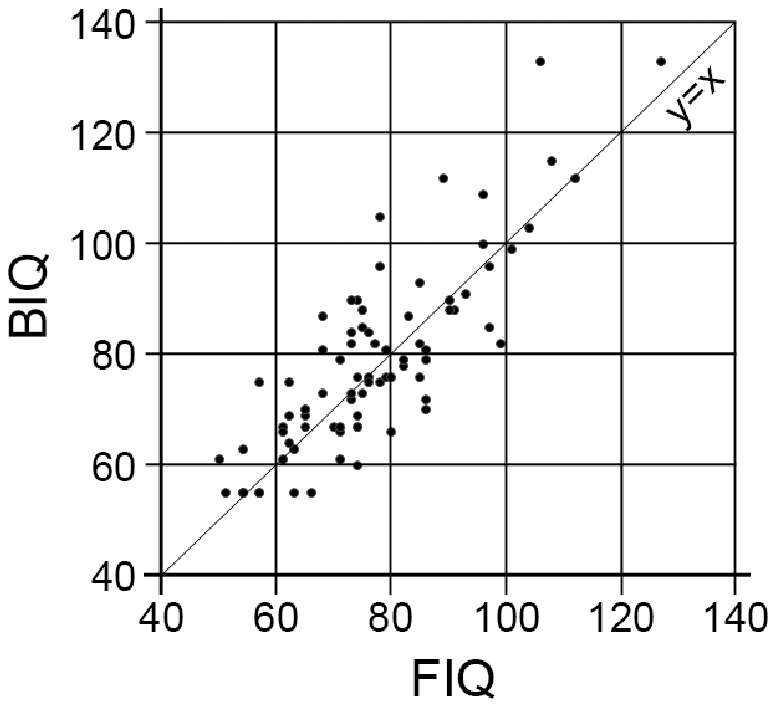
Distribution of BIQ and FIQ scores. Notes: The straight line represents the diagonal line y = x.

Figure 1 shows the distributions of the FIQ and BIQ scores. The mean FIQ was 76.5 (SD 15.0), while the mean BIQ was 78.5 (SD 16.9). The mean difference was 2.0 (SE 1.0, 95% CI −4.1–0.1). The MPE ± SE was 0.03±0.01, and the RMSE was 0.13. There was little deviation of the BIQ from the FIQ.

### Validity and Clinical Utility

#### ANOVA ICC

The ANOVA ICC between the BIQ and FIQ was very high (0.83). Additionally, the ICC between BIQ with both the WISC-III verbal IQ (VIQ) and the performance IQ (PIQ) were also high (correlation coefficients: ICC = 0.72 and 0.81, respectively). Neither the VIQ alone nor the PIQ alone was predominantly reflected (see Table 1).

**Table 1:** Mean IQs and Intraclass correlation coefficients between BIQ and each of the WISC-III IQs.

	Mean (S.D.)	ICC between BIQ and each IQ
**FIQ**	76.5 (15.0)	0.83
**VIQ**	79.0 (14.2)	0.72
**PIQ**	78.8 (15.7)	0.81
**BIQ**	78.5 (16.9)	–

Notes. ICC, intraclass correlation coefficient; BIQ = The new Tanaka B Intelligence Scale IQ; FIQ = Full IQ; VIQ = Verbal IQ; PIQ = Performance IQ.

#### ROC analysis and the SSLRs

**Figure 2:**
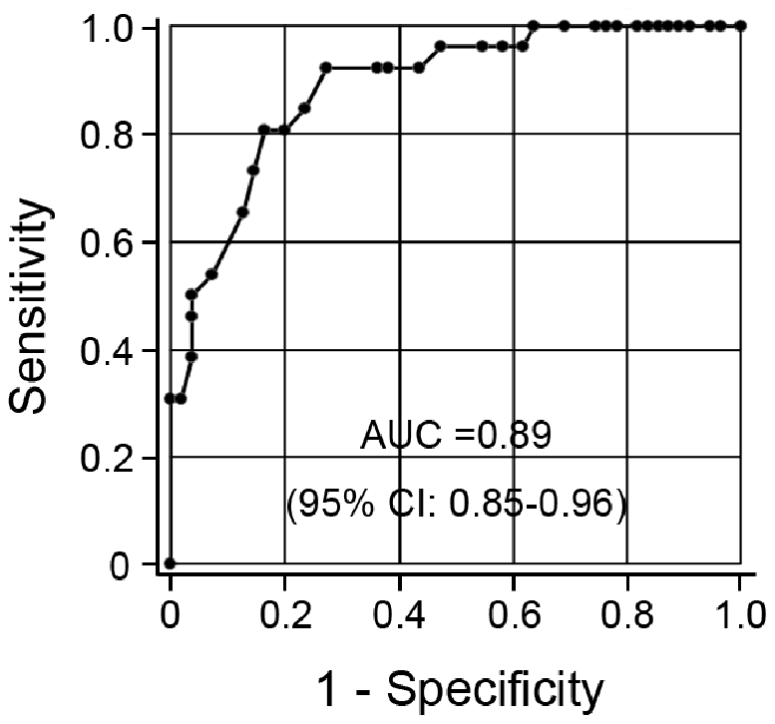
Receiver operating characteristic curves for BIQ for intellectual disability according to the WISC-III. Notes: AUC, area under the curve; 95% CI, 95% confidence interval.

ROC analysis showed that the BIQ enabled screening of FIQ<70 with a high discrimination ability for FIQ<70 (area under the curve (AUC) = 0.89, 95% CI: 0.85–0.96) (see Fig. 2).

Next, the SSLRs were calculated. For the BIQ 51–60 stratum and the BIQ 61–65 stratum, the SSLRs were ≥10 (post-test probability for each stratum: 89%, 83%, respectively). Thus, individuals who have a deficit in intellectual function could be identified as possible. In addition, for the BIQ 76–85 stratum and the BIQ≥86 stratum, the SSLRs were approximately 0.1 (post-test probability of both strata: 5%). Thus, individuals who have a deficit in intellectual function could also be ruled out. For the BIQ 66–70 and 71–75 strata, the SSLRs were 2.4 and 1.1, respectively; the post-test probabilities were 33% and 53%, respectively (see Table 2). In the BIQ≤65 group overall, the SSLR was 13.8 (95% CI: 3.9–48.9, post-test probability: 87%); in the BIQ≥76 group overall, the SSLR was 0.11 (95% CI: 0.03–0.4, post-test probability: 5%).

**Table 2:** Stratum-specific likelihood ratios and posttest probabilities of the BIQ for FIQ<70.

BIQ Stratum	Subjects		SSLR (95% CI)	Posttest probability
	FIQ≥70	FIQ<70		
51–60	1	8	16.9 (3.2–90.2)	0.89
61–65	1	5	10.6 (1.9–60.6)	0.83
66–70	7	8	2.4 (1.0–5.8)	0.53
71–75	6	3	1.1 (0.3–3.6)	0.33
76–85	19	1	0.1 (0.02–0.6)	0.05
≥86	21	1	0.02 (0.02–0.5)	0.05

Notes. SSLR, Stratum-specific likelihood rations; 95% CI, 95% confidence interval; BIQ = The new Tanaka B Intelligence Scale IQ; FIQ = Full IQ.

## Discussion

The new Tanaka B Intelligence Scale, an intelligence test that can be administered on groups, has been shown to have high split-half correlations, test-retest reliability, and concurrent validity with academic performance. However, there has not been enough information about this test for use as a standardized intelligence test. To standardize the new Tanaka B Intelligence Scale, the present study examined the reliability of the test and its concurrent validity with the WISC-III, an already established and standardized test for individual testing. Additionally, the clinical utility of the new Tanaka B Intelligence Scale was considered. Using the new Tanaka B Intelligence Scale in subjects aged ≥14 years old, there was high internal consistency and concurrent validity with the WISC-III. This demonstrated that, even in settings where performing individual intelligence tests (e.g. the WISC-III) is difficult, the new Tanaka B Intelligence Scale, a group intelligence test can be easily performed, can assess overall intellectual development and become one of the alternative to an individual test such as the WISC-III.

With an IQ score of the new Tanaka B Intelligence Scale≤65, the SSLR was ≥10 (post-test probability: 87%), and in the BIQ≥76 strata, the SSLRs were approximately 0.1 (post-test probability: 5%). Therefore, individuals who have FIQ<70 could be ruled in or out. In other words, ID can be efficiently diagnosed using detailed intelligence tests in individuals with a BIQ range of 66–75. Thus, this may be a useful test to easily screen for ID in the future.

The new Tanaka B Intelligence Scale can be administered on groups within a short period of time, with no special equipment or training required. Therefore, it can be performed in a variety of settings, enabling expanded assessment of intellectual development, even in locations where administering the WISC-III has previously been difficult. Furthermore, the verbal exchanges are simple instructions, and no complex interaction is required. Additionally, testing can be conducted on individuals with various linguistic backgrounds and verbal levels. In the present study, aside from a single cut-off point, by calculating SSLRs, predicted post-test probability for the results obtained could be determined. This point is important and significant in terms of clinical usefulness.

The SD of the FIQ for subjects in the present study was 15.0, so that the overall variation was normal. The range in intelligence was an FIQ of 51–127, thus covering the approximate strata for the general intelligence category and the category requiring an estimate of deficit in intellectual function. Furthermore, there was little work-up bias or spectrum bias in the juvenile detention home. However, the mean IQ was low, at 76.5±15.0, and 32.1% of the sample had an FIQ<70. The mean IQ of residents in juvenile correctional systems is lower than the IQ in the general public [27]–[33], which probably had an effect. However, none of the subjects had moderate to severe deficit in intellectual function, such as an FIQ≤50. Therefore, although there was some sample bias, many subjects had an IQ near the borderline for deficit in intellectual function, which was also an advantage in this study. Future studies should include a broader range of subjects. In addition, 95.1% of the subjects were male, therefore future studies may also want to investigate the influence of sex. However, previous studies have reported that sex differences in VIQ, PIQ, and FIQ were negligible in Japanese and American samples [34].

In this study, the ratios of individuals who were diagnosed as having psychiatric disorders other than ID; such as Attention Deficit/Hyperactivity Disorder, were not significantly high compared with prevalence of these disorders in the general population. Therefore, sample bias on this point might be negligible. On the other hand, in terms of delinquent behavior, there might be sample bias, because most cases in this study conducted, or were entangled in, a delinquent act.

In conclusion, this study demonstrated sufficient reliability and concurrent validity of the new Tanaka B Intelligence Scale, a group intelligence test. In addition, the clinical utility of the scale in screening for individuals who have a deficit in intellectual function was also demonstrated. The validity of this test should be further evaluated within a broader setting including a wider range of subjects, for example, using a randomized sample of the general population. Additionally, the new Tanaka B Intelligence Scale may be performed on many different cultures, since it is easy to conduct, has simple instructions, and is not influenced by strong barriers to language. It is hoped that the present study’s results contribute to the proper assessment of intellectual development as well as specialized and effective care and services based on the current findings.
